# Controlling the Spread of HIV/AIDS in the Indian Subcontinent

**DOI:** 10.1371/journal.pmed.0020420

**Published:** 2005-12-27

**Authors:** Govindasamy Agoramoorthy, Minna J Hsu

**Affiliations:** **1**Tajen UniversityYanpu, TaiwanRepublic of China; **2**National Sun Yat-sen UniversityKaohsiung, TaiwanRepublic of China

The article on HIV/AIDS infection by Singh and colleagues outlines an alarming fact about the spread of this deadly virus in Nepal [1]. We would like to add that more assertive campaigns are necessary to curb the spread of infection in the Indian subcontinent before it's too late. In the year 2000 alone, a total of 5.3 million people were infected with HIV worldwide [2]. Since the epidemic started two decades ago, HIV/AIDS has killed 22 million people globally. India, Indochina, and the former Soviet republics have seen the most rapid rise of HIV incidence in recent years. AIDS experts have raised alarm bells over its spread in the Asia-Pacific region, and called for a united effort to control it. The Joint United Nations Programme on HIV/AIDS (UNAIDS) estimates about 5 million people in India alone are infected.

The first report of HIV/AIDS infection in India was in 1986, and since then the virus has spread rapidly throughout the country. Both HIV serotypes 1 and 2 exist in India, and HIV-1 C is the most common subtype reported. Sexual transmission of HIV is the predominant route of transmission in India [3]. According to the Ministry of Health in New Delhi, only 3% of Indians use condoms for birth control, since the tradition and culture dictate that women undergo sterilization or take birth control pills. Prostitution plays a major role in spreading the disease among the heterosexuals in urban areas. Although Mumbai appears to be the main locus for AIDS, rapid spread has occurred through other major cities as well. Migration of people from cities to rural areas is so rapid that the disease may already be out of control in many areas. The blood screening tests conducted at most hospitals in rural India are not adequate to confirm presence of the virus, making blood transfusion unsafe. The National AIDS Control Organization (NACO), the apex body for controlling AIDS in India, has reported a high incidence (8.2%) of blood donors who are HIV-positive among healthy blood donors in urban areas [4].

AIDS is a sexually transmitted disease, and as long as people are educated thoroughly and warned about the dangerous consequences of unsafe sex, there is less to fear. Unfortunately, the intervention program launched by the NACO had very little impact in controlling the spread of the epidemic in India [4]. The current educational programs are often restricted to the passive dissemination of information through posters, media, and display of safe-sex billboards behind automobiles. More aggressive efforts are, therefore, needed to reach out to each and every rural/urban community throughout India to combat the spread of the disease. The state and central government agencies must build specialized shelters for people with HIV/AIDS. More funds must be spent for effective AIDS awareness campaigns, research, routine screening tests, and treatment.

According to the Asia Pacific Network of People Living with AIDS, a considerable number of people were refused treatment or delayed provision of treatment or health services after being diagnosed with HIV/AIDS. Breaches of confidentiality by health workers were common in Asian countries. Within families and communities, women were discriminated against more than men—including ridicule, harassment, and physical assault—and they were often forced to change their place of residence because of their HIV status [5].

Although politicians and policymakers are increasingly committed to AIDS prevention and control efforts in countries such as India, a multidisciplinary approach such as early identification and treatment of sexually transmitted diseases, promotion of condom usage, rapid blood screening to test for HIV in rural areas, public awareness campaigns, poverty eradication, and development of prevention interventions have to be considered for effective control of the spread of this virus in the Indian subcontinent.

Moreover, people from all walks of life must take an active role to promote AIDS awreness and prevention across the Indian subcontinent. It is time for the local and regional celebrities, such as political leaders, movie stars, and beauty pageant winners, in the Indian subcontinent to get involved in helping people with HIV and in educating the public, which would certainly raise awareness among the rural public more quickly than current efforts. It is time to remember how the late Princess of Wales reached out to people with AIDS, shook hands to console them, and also raised millions of dollars for their welfare. Countries in the Indian subcontinent have experienced and handled the outbreak of deadly epidemics in the past [6], and we hope that AIDS can also be controlled and eradicated eventually in the near future.
